# Bioprospecting of Coralline Red Alga *Amphiroa rigida* J.V. Lamouroux: Volatiles, Fatty Acids and Pigments

**DOI:** 10.3390/molecules26030520

**Published:** 2021-01-20

**Authors:** Ana-Marija Cikoš, Ivana Flanjak, Krunoslav Bojanić, Sanja Babić, Lara Čižmek, Rozelindra Čož-Rakovac, Stela Jokić, Igor Jerković

**Affiliations:** 1 Department of Process Engineering, Faculty of Food Technology, Josip Juraj Strossmayer University of Osijek, Franje Kuhača 18, 31000 Osijek, Croatia; acikos@ptfos.hr; 2 Department of Food and Nutrition Research, Faculty of Food Technology, Josip Juraj Strossmayer University of Osijek, Franje Kuhača 18, 31000 Osijek, Croatia; ivana.flanjak@ptfos.hr; 3 Laboratory for Biotechnology in Aquaculture, Division of Materials Chemistry, Ruđer Bošković Institute, Bijenička cesta 54, 10000 Zagreb, Croatia; Krunoslav.Bojanic@irb.hr (K.B.); Sanja.Babic@irb.hr (S.B.); Lara.Cizmek@irb.hr (L.Č.); Rozelindra.Coz-Rakovac@irb.hr (R.Č.-R.); 4 Department of Organic Chemistry, Faculty of Chemistry and Technology, University of Split, Ruđera Boškovića 35, 21000 Split, Croatia

**Keywords:** red algae, extraction, volatile compounds, fatty acids, pigments, antioxidant activity

## Abstract

Due to the lack of phytochemical composition data, the major goals of the present study on *Amphiroa rigida* J.V. Lamouroux were to: (a) investigate and compare volatilome profiles of fresh and air-dried samples obtained by headspace solid-phase microextraction (HS-SPME) and hydrodistillation (HD) followed by gas chromatography and mass spectrometry (GC/MS) analysis; (b) determine fatty acids profile by gas chromatography with flame ionization detector (GC-FID); (c) obtain the pigment profiles of semipurified extracts by high performance liquid chromatography (HPLC) and (d) evaluate the antioxidant and antimicrobial activities of its less polar fractions. The comparison of headspace of fresh (FrAr) and air-dried (DrAr) samples revealed many similarities regarding the presence and abundance of the major (heptadecane and pentadecane) and minor compounds. The hydrodistillate (HD) of DrAr profile was quite different in comparison to HD-FrAr. The predominant compound in HD-FrAr was (*E*)-phytol. In HD-DrAr, its percentage was approximately one-half reduced, but the abundance of its degradation product phytone and of unsaturated and oxygenated compounds increased indicating more intense fatty acid decomposition and oxidation during drying. The fatty acid determination revealed that the most dominant was palmitic acid (42.86%) followed by eicosapentaenoic acid (19.14%) and stearic acid (11.65%). Among the pigments, *A. rigida* contained fucoxanthin (0.63 mg g^−1^ of dry fraction), lutein (5.83 mg g^−1^), β-carotene (6.18 mg g^−1^) and chlorophyll *a* (13.65 mg g^−1^). The analyzed less polar fractions of *A. rigida* exhibited antioxidant scavenging activity with diammonium salt of 2,2′-azino-bis (3-ethylbenzthiazolin-6-yl) sulfonic acid (ABTS) assay up to 3.87 mg g^−1^ trolox equivalents (TE), and with the oxygen radical absorbance capacity (ORAC) assay up to 825.63 μmol g^−1^ TE (with carotenoids as the major contributors).

## 1. Introduction

In marine ecosystems, algae can release a wide spectrum of volatile organic compounds (VOCs) with ecological functions to increase the tolerance to abiotic stresses, transfer stress information to induce defense, play allelopathic roles or protect against the predators [1]. Macroalgae are known for their content of long-chain polyunsaturated fatty acids (PUFAs) such as omega-3 fatty acids that are associated with the several biological activities and health benefits including antioxidant [2], antibacterial, antitumor and anti-inflammatory effects [3]. Red algae, especially species belonging to the order Corallinales, are rich in eicosapentaenoic acid (EPA) that is known as omega-3 essential fatty acid [4]. The pigments are characteristic for certain algal groups (Chlorophyta, Rhodophyta and Ochrophyta). Red algae are known for their content of chlorophyll *a*, lutein, zeaxanthin, α-carotene and β-carotene and phycobilin pigments that are responsible for red or pink color of this phylum and enable them to grow in deep waters [5]. Numerous studies discovered that carotenoids are known for their powerful antioxidant [2,6], anticancer [7] and antibacterial activities [8].

In general, there are just a few papers on red alga *A. rigida*. Mostly taxonomic studies on *Amphiroa* species were done [9,10,11,12] and the morphological variability of *A. rigida* was investigated [13,14]. The external microscopic characters obtained with the scanning electron microscope (SEM) were illustrated: the organization of intergenicular surfaces; differentiation and superficial aspects of conceptacles and quantitative characters such as epithallial concavity diameter and variation in the thickness of calcification [14]. However, according to our best knowledge, there is only one recent phytochemical study on this alga reporting polysaccharides. The novel polysaccharides from *A. rigida* (ARPS) were extracted [15] by an ultrasonication disruption (USD) and the extraction conditions were optimized by response surface methodology (RSM) combined with Box–Behnken design (BBD). Furthermore, ARPS exhibited great potential for in vitro antioxidant activity in DPPH (58.24% ± 0.65%) and diammonium salt of 2,2′-azino-bis (3-ethylbenzthiazolin-6-yl) sulfonic acid (ABTS^+^) scavenging (52.23% ± 0.88%) activity and also showed good antibacterial activity against *Salmonella typhi*. In addition, ARPS can inhibit human breast cancer cells (MCF-7) [15]. Hence, there are some studies reporting activities of other species belonging to the genus *Amphiroa* such as antioxidative and matrix metalloproteinase inhibitory effects of *A. dilatata* [16], antimicrobial activity of unspeciated *Amphiroa* algae [17] and antifungal and antibacterial activities of *A. bowerbankii* and *A. ephedraea* [18]. The fatty acids profiles are reported for *A. anceps* [19] and *A. beauvoisii* [20] with some differences among profiles but it may be due to the different geographical locations and diverse abiotic factors.

Due to the lack of literature data with the phytochemical composition of marine macroalgae from the Adriatic Sea we decided to provide the first detailed phytochemical study toward targeted compounds from red alga *A. rigida* J.V. Lamouroux collected from the Adriatic Sea. This is the first report on the headspace and volatile oil composition including both fresh (FrAr) and air-dried (DrAr) samples of *A. rigida* isolated by hydrodistillation (HD) and headspace solid-phase microextraction (HS-SPME). In addition, the fatty acids and pigments profiles were determined in freeze-dried sample of *A. rigida* (FdAr) for the first time and FdAr non-polar fractions were evaluated with respect to antimicrobial activity and antioxidant scavenging ability. The main goals of the present study on *A. rigida* were to: (a) investigate and compare volatilome profiles of FrAr and DrAr obtained by HS-SPME and HD followed by gas chromatography with flame ionization detector and mass spectrometry (GC-FID/MS) analysis; (b) determine fatty acids profile of FdAr after derivatization as methyl esters by GC-FID analysis; (c) evaluate pigment profiles of semipurified less polar fractions of FdAr by high performance liquid chromatography (HPLC); (d) get an insight of antimicrobial activity of semipurified less polar fractions of FdAr against chosen bacterial and fungal panels; (e) evaluate the antioxidant activity of semipurified less polar fractions of FdAr by two assays, the oxygen radical absorbance capacity (ORAC) and the reduction of radical cation ABTS^•+^ and (f) expand the knowledge of taxonomically related similarities/differences/chemical markers in the chemistry of marine red algae.

## 2. Results and Discussion

### 2.1. Analysis of Volatile Organic Compounds (VOCs) of A. rigida

A very small number of the Adriatic Sea algae have been investigated for the presence of VOCs. HS-SPME and HD were applied as complementary techniques to obtain the full profile of the alga VOCs [21,22,23]. HS-SPME is suitable for determining the headspace VOCs in the algae with the following advantages: small sample requirement, minimal sample handling, single-step gas extraction, quickness and solvent free. To obtain more detail headspace profiles two fibers of different polarities were used for HS-SPME: PDMS/DVB and DVB/CAR/PDMS. HD is used for obtaining the volatile oils from the algae without the interference of non-volatile compounds that are not isolated. The chromatographic analysis by GC-FID/MS was used. Striking differences were found between the obtained HS-SPME and HD chemical profiles.

#### 2.1.1. The Headspace Composition of *A. rigida*

The GC–MS survey indicates the presence of two normal odd alkanes (heptadecane (C_17_) up to 47.77% and pentadecane (C_15_) up to 7.08%) covering the majority of total headspace profile of fresh *A. rigida* (HS-FrAr). Air-dried sample also contained in the headspace (HS-DrAr) C_17_ as the major constituent (up to 53.96%) followed by C_15_ (up to 7.02%). Only one more saturated hydrocarbon (eicosane, C_20_) was found as the minor constituent of HS-FrAr (Table 1). Other abundant compounds (with the percentages from 3 to 4 or more orders of magnitude below those of C_17_) in HS-FrAr were unsaturated hydrocarbons (Table 1): pentadec-1-ene up to 13.05%, (*E*)-pentadec-7-ene up to 6.00%, hexadec-1-ene up to 11.25% (*E*)-heptadec-3-ene up to 4.79%, (*E*)-heptadec-8-ene up to 0.77% and heptadec-1-ene up to 1.04%. Their abundance in HS-DrAr was slightly changed and C_15_-alkenes were not present (Table 1). Ectocarpene (6-[(*Z*)-but-1-enyl]-cyclohepta-1,4-diene) was only identified cyclic unsaturated hydrocarbon found in HS-FrAr, but in low abundance. Saturated and olefinic hydrocarbons were already found in different species of marine algae [24,25]. It was previously found that the major hydrocarbons of red algae *Porphyridium cruentum*, *Gigartina* sp. and *Plocamium* sp. were more highly saturated, particularly the straight-chain hydrocarbons C_17_ and C_15_ [24,26]. Heptadecane is strongly predominant in red alga *A. rigida* headspace supporting previous findings on red algae [24,25,27]. The investigations of the elongation–decarboxylation pathway in the algae using radiotracers [28] detected decarboxylases capable of converting fatty acid to *n*-alkane and decarboxylation of stearic acid to heptadecane in the alga *Anacystis nidulans* was determined by ^13^C- and ^2^H-labeling and ^13^C nuclear magnetic resonance [29]. According to that, possible explanation of predominance of heptadecane in the headspace of *A. rigida* may be the degradation of stearic acid, which was detected in *A. rigida* in present study. Several isomeric heptadecenes are present in the benthic marine algae [25] and a predominance of heptadec-3-ene and heptadec-7-ene was suggested in brown and red algae, which is in partial agreement with the current findings.

Another group of present aliphatic compounds in HS-FrAr consisted of lower saturated aldehydes pentanal (up to 1.25%), hexanal (up to 6.72%), heptanal and nonanal (up to 1.63%) followed by unsaturated alkenals (*E*)-pent-2-enal, (*E*)-hex-2-enal (up to 7.41%), (*E*,*E*)-hepta-2,4-dienal, (*E*)-oct-2-enal (up to 0.69%) and (*E*)-dec-2-enal. Among them only C_16_-, C_17_- and C_18_ -aldehydes and (*E*)-hex-2-enal were identified in HS-DrAr. Of the identified aldehydes hexanal was the major compound in HS-FrAr and HS-DrAr similar as in other algae [30,31]. The seaweeds aldehydes may be derived from the degradation of PUFAs, either by autooxidation or by the enzymatic action of lipoxygenases [32], for example hexanal can be formed from linoleic acid via sequential action of lipoxygenase/fatty acid hydroperoxide lyase [33,34]. In our study linoleic acid was detected in *A. rigida*. Generally, red algae that showed decreased concentrations of PUFAs showed higher concentrations of aldehydes indicating degradation of fatty acids [27]. These aldehydes are widespread, and have already been found in many other sea products, such as green and brown macroalgae [35,36], lobsters [37] and oysters [38]. Some of them (e.g., unsaturated aldehydes C_7_, C_8_ and C_10_) play a role as defense agents such as the inhibition of egg cleavage of sea urchins and reduction of hatching success in copepods [39]. Several aliphatic alcohols and alkenols were found in HS-FrAr: pentan-1-ol, (*Z*)-pent-2-en-1-ol, hexan-1-ol (up to 2.36%), oct-1-en-3-ol (up to 0.5%), octan-1-ol (up to 0.49%) and tetradecan-1-ol (up to 1.02%). Among them only oct-1-en-3-ol was present in HS-DrAr. The aldehydes can be converted either enzymatically or spontaneously to other alcohols or alkenols. 6-Methylhept-5-en-2-one was present up to 0.67% in HS-FrAr and up to 0.90% in HS-DrAr. It was the most abundant ketone and it is common compound in the algae (e.g., it was found in relatively high amount in the green alga *Capsosiphon fulvescens* [40]). 6-Methylhept-5-en-2-one was the only aliphatic ketone present in HS-FrAr and HS-DrAr, while octan-2,3-dione and (*E*,*E*)-octa-3,5-dien-2-one were present only in HS-DrAr.

Degraded carotenoid products were also present: (*E*)-β-ionone in HS-FrAr (up to 1.55%) and HS-DrAr and β-cyclocitral only in HS-FrAr. It is known that β-ionone is formed by the enzymatic cleavage of β-carotene [41] so that it implies that the β-ionone in *A. rigida* may be formed from β-carotene that was found in our study. Those two compounds are ubiquity found in marine algae (e.g., in cyanobacteria [42]). In the later stages of carotenoid degradation (further oxidation of longer chain intermediates), increasing amounts of short-chain mono and dioxygenated compounds including β-cyclocitral are formed [43]. β-Cyclocitral, α-ionone and β-ionone showed inhibitory effects on *C. pyrenoidosa* cell growth [44]. β-Cyclocitral can cause cell rupture of *Nitzschia palea* [45] and high concentration β-cyclocitral even impact the growth of *Microcystis* by causing lysis [46].

Among other constituents, two benzene derivatives were found (benzaldehyde in HS-FrAr (up to 1.60%) and HS-DrAr (up to 1.16%) and benzyl alcohol exclusively in HS-DrAr (up to 9.44%)) and three sesquiterpenes (germacrene D in HS-FrAr and HS-DrAr, β-bourbonene and α-cubebene in HS-FrAr). In addition, tribromomethane up to 1.52% was found only in HS-FrAr and it was found previously in *Ascophyllum nodosum* [47] and *Halopteris filicina* [13,23]. Marine macroalgae possess a high ability to fix halide ions by the action of haloperoxidase enzymes and in the presence of hydrogen peroxide, these ions were oxidized and then could react with organic substrates [48].

Summary comparison of HS-FrAr and HS-DrAr revealed many similarities (Table 1) regarding the presence and abundance of the major compounds (e.g., C_17_, C_15_, hexanal, (*E*)-hex-2-enal and (*E*)-heptadec-3-ene) as well for many minor constituents (e.g., heptanal, oct-1-en-3-ol, 6-methylhept-5-en-2-one, (*E*)-oct-2-enal, nonanal and benzaldehyde). However, several compounds were found exclusively after air-drying (such as 2-pentylfuran, benzyl alcohol, octan-2,3-dione, (*E*)-octa-3,5-diene-2-one and decanal) indicating higher accumulation of oxidation products in HS-DrAr. The most striking difference was high benzyl alcohol percentage (up to 9.44%) found exclusively in HS-DrAr that could origin from benzaldehyde or other parent compounds during air-drying. Dimethyl sulfide (DMS) appeared in HS-DrAr and it is the algal osmolyte, which derives from dimethylsulfoniopropionate (DMSP) that has been synthetized and accumulated in a wide range of taxa [49]. In addition, 2-pentylfuran was only found in HS-DrAr indicating oxidative degradation of linolenic acid [50] or sugar fragmentation by Maillard reactions [51].

#### 2.1.2. Volatile Oil Composition of *A. rigida*

The predominant compound in the hydrodistillate of fresh *A. rigida* (HD-FrAr) was (*E*)-phytol (41.75%) accompanied with a smaller abundance of its degradation product phytone (Table 2). Diterpene alcohol (*E*)-phytol is a ubiquitous compound in chlorophyll *a* containing marine algae [52] and was already found in the volatile oil of different green algae [21,53]. The red algae are known for their content of chlorophyll *a,* which is present in higher amounts than in green algae [54]. Detected (*E*)-phytol could be formed from chlorophyll *a* degradation, which was detected in our study. Phytol oxidation could lead, among others, to methylated long chain fatty acid ketone—hexahydroxyfarnesyl acetone (6,10,14-trimethylpentadecan-2-one, phytone) that was found in the oil from the dried sample (HD-DrAr) at 7.28% and HD-FrAr at 2.21%. Phytone results mainly from the hydrolysis of chlorophyll or bacteriochlorophyll-a photoproducts or from phytol biodegradation [55].

Two hydroazulene diterpenes were found in HD-FrAr and HD-DrAr: pachydictol A (2.97%; 1.11%) and isopachydictyol A (2.41%; 1.75%). Both of these hydroazulene diterpenes were found previously in marine algae [56]. Diterpene alcohol epimanool was present in HD-FrAr (3.18%) and HD-DrAr (1.77%) while diterpene of abietane chemical class ferruginol was found in HD-FrAr (1.45%). Several sesquiterpenes (β-bourbonene, germacrene D, cubebol, *trans*-farnesol and eudesma-4(15),7-dien-1-β-ol) were present in HD-FrAr and HD-DrAr as minor constituents (Table 2), while cubenol appeared only in HD-DrAr. All these compounds were not present in the headspace, probably due to lower volatility.

Alkanes C_17_ and C_15_, the major compounds of HS-FrAr and HS-DrAr, were present in HD-FrAr and HD-DrAr, but at more than ca. 5-10 orders of magnitude lower percentages (3.80% and 12.22%; 2.36% and 0.91%, respectively) in comparison to the headspace. Several higher aliphatic alkenes were present (the same ones as found by HS-SPME) as minor constituents: pentadec-1-ene, (*E*)-pentadec-7-ene, (*E*)-heptadec-8-ene and (*E*)-heptadec-3-ene. Saturated and unsaturated carbonyl compounds were also identified such as (*E*)-hex-2-enal, heptanal, 6-methylhept-5-en-2-one, octan-2-one, octanal, (*E*)-oct-2-enal, nonan-2-one, nonanal, undecan-2-one, pentadecanal and heptadecan-2-one. Few aliphatic alkanols and alkenols were identified; 2-ethylhexan-1-ol, octan-1-ol, dodecan-1-ol, tridecan-1-ol, pentadecan-1-ol, (*E*)-hexadec-11-en-1-ol and hexadecan-1-ol.

Two benzene derivatives were found in HD-FrAr and HD-DrAr: benzyl alcohol and 2-phenylacetaldehyde.

In summary, HD-DrAr profile was quite different in comparison to HD-FrAr. The percentage of (*E*)-phytol was double reduced up to 16.44%, but at the same time the abundance of phytone increased to 7.28%. Heptadecane was increased to 12.22% and (*E*)-heptadec-8-ene, (*E*)-heptadec-3-ene and pentadec-1-ene indicating more intense fatty acid decomposition. The abundance of oxygenated compounds (e.g., (*E*)-hex-2-enal, heptanal, octan-2-one, octanal, (*E*)-oct-2-enal, octan-1-ol, nonanal, decan-2-one, pentadecanal, pentadecan-1-ol, hexadecan-1-ol and heptadecan-2-one) was also elevated indicating oxidation reactions. More intensive oxidation and fatty acid decomposition were confirmed by the formation of additional oxygenated compounds, not found in the headspace such as: heptan-2-one, (*E*)-hept-2-enal, oct-1-en-3-ol, octane-2,3-dione, (*E*,*Z*)-octa-3,5-dien-2-one, (*E*,*E*)-octa-2,4-dienal, (*E*,*Z*)-nona-2,6-dienal, (*Z*)-non-2-enal, decanal, decan-1-ol, (*E*,*Z*)-deca-2,4-dienal, (*E*,*E*)-deca-2,4-dienal, undecan-1-ol, dodecan-1-ol, tridecanal, tetradecanal, (*Z*)-hexadec-11-en-1-ol and octadecanal. Additionally, degradation of carotenoids was promoted since the percentage of (*E*)-β-ionone was elevated and β-cyclocitral was only present in HD-DrAr.

### 2.2. Fatty Acids Composition of A. rigida

Fatty acids composition of *A. rigida* is presented in Table 3. After GC-FID analysis 14 fatty acids were identified in *A. rigida.*

As shown in Table 3, the main fatty acids were palmitic acid (C16:0), as the dominant one, followed by eicosapentaenoic acid (EPA, C20:5n3) and stearic acid (C18:0) with mean values 42.86%, 19.14% and 11.65%, respectively. The content of saturated fatty acids (SFA) was 59.65% followed by a considerable amount of PUFAs, 33.16%. PUFAs are important essential components that play an important role in several human pathologies (e.g., obesity, diabetes mellitus type 2, heart diseases and atherosclerosis). Generally, phylum Rhodophyta is characterized for containing a large diversity of essential fatty acids, especially orders Gigartinales, Corallinales and Gracilariales. Algae belonging to these orders are rich in EPA and arachidonic acid (C20:4n6) [3,57]. Given the relatively high content of EPA in this red algae, there is a possibility of using this alga as a good source of essential omega-3 fatty acids, but further research for investigation of their bioavailability is necessary. *A. rigida* contained a significantly higher amount of EPA (19.14%) when compared to other red algae examined in a recent study [27] with the values ranging from 0.47% to 7.95%. Jayasree et al. [19] examined fatty acid composition of *Amphiroa anceps* and palmitic acid was shown to be the major component with a mean value of 57.57%, while arachidonic acid was the second abundant compound (18.67%). Regarding the amount of EPA, it was significantly lower when compared to our study (2.46%). On the other hand*, Amphiroa beauvoisii* contained higher amounts of EPA (25.2%). These variations among species belonging to the same genus can be explained by the exposure to diverse abiotic factors, and by different geographical locations resulting in different fatty acids profiles [20]. We did not find arachidonic acid in *A. rigida,* which was not in accordance with previous studies indicating that the algae belonging to phylum Rhodophyta contained high concentrations of that fatty acid [58,59].

### 2.3. Pigment Composition of A. rigida

HPLC analysis of the pigments was performed on semipurified fractions 3 (F3) and 4 (F4) of FdAr with the solvents MeOH and MeOH:DCM (1:1, *v*/*v*) after the removal of water soluble components that were eluted in the fractions 1 (F1; H_2_O) and 2 (F2; MeOH:H_2_O, 1:1, *v*/*v*). Generally, used solvents, MeOH and MeOH:DCM, were shown as suitable for the extraction of lipophilic pigments, such as chlorophylls and carotenoids that are the main groups of pigments found in the algae [60,61]. Different pigments were detected considering the applied solvent as can be seen in Table 4. Fucoxanthin was detected in F3 with the amount of 0.63 ± 0.62 mg g^−1^, and lutein, which was the main peak in this fraction, with the concentration of 5.83 ± 0.97 mg g^−1^, while these two pigments were not detected in F4 with used less polar mixture of solvents (MeOH:DCM). Hence, F4 revealed the presence of chlorophyll *a* with the amount of 13.65 ± 0.50 mg g^−1^ and β-carotene with the concentration of 6.18 ± 0.72 mg g^−1^. Additionally, α-carotene was detected but the concentration was under the limit of quantification. Even though, α-carotene and β-carotene are known as the major carotenoids present in red algae, we did not detect α-carotene at a high concentration. On the other hand, lutein was detected and it was suggested that it is formed by the hydroxylation of α-carotene [62].

Kavalappa et al. [7] used similar mixture of solvents (MeOH:DCM:H_2_O) for the extraction of pigments from red algae *Gracillaria* spp. Among all tested macroalgae, red alga *Gracillaria* spp. showed a dominance of β-carotene and lutein as the major carotenoids, which was also shown in our study. Generally, the presence of β-carotene and lutein is characteristic for red algae, especially for the order Corallinales where *A. rigida* also belongs. There are contrary reports of the pigment composition of red algae and it is very difficult to find the relationship between the pigment profile and phylogeny of red algae. Some reports indicate that the main xanthophylls in red algae are zeaxanthin and lutein [63], while others reported more complex carotenid profiles including fucoxanthin and neoxanthin [64]. In our study we found lutein and fucoxanthin, which was not in accordance with mentioned studies pointing out high diversity of pigment profiles among red algae. Explanation for the lacking of some pigments, such as zeaxanthin, which is characteristic for red algae, can be related to the capability to synthesize this pigment and photoacclimation state of the algae. Schubert et al. [64] did not detect zeaxanthin in organisms that were collected from the low tidal zone, while it was detected in species from the high tidal zone. Additionally, investigated algae from the order Corallinales showed the presence of lutein as the major carotenoid and zeaxanthin with minor concentration or completely missing. This may explain why we did not detect zeaxanthin in the fractions of *A. rigida* and lutein was showed as the major carotenoid in F3. The only ubiquitous pigments in all analyzed red algae were α-carotene and β-carotene [64] that was also shown in our study. Furthermore, the presence of fucoxanthin can be associated with epiphyte contamination of the samples [63]. According to the literature [54], among chlorophylls (*a, b, c* and *d*) only chlorophyll *a* is present in red algae, which was also shown in our study. It is suggested that the content of chlorophyll *a* in red algae depends on available sunlight. Namely, in the months with the lowest sunlight chlorophyll *a* is present with the highest amounts [65], which can explain the high concentration of chlorophyll *a* found in *A. rigida*. Chlorophyll *a* was present in higher amount in *A. rigida* when compared to the amount found in *Amphiroa fragilissima,* while the content found in another red alga, *Gracilaria foliifera,* [66] was very similar to the content obtained in our study.

### 2.4. Antimicrobial Activity of A. rigida

Antimicrobial activity of *A. rigida* semipurified less polar fractions, F3 and F4, was evaluated against the bacterial panel including *Escherichia coli, Pseudomonas aeruginosa, Staphylococcus aureus, Bacillus subtilis* subsp. *spizizenii, Listonella (Vibrio) anguillarum, Photobacterium damselae* subsp. *damselae* and *P. damselae* subsp. *piscicida* and against the fungal panel including *Candida albicans* and *Fusarium oxysporum* f. sp. *Lagenariae*. The fractions, F3 and F4, did not show antimicrobial activity against any indicator species used when tested with broth microdilution (400–0.78 µg mL^−1^ (*w*/*v*)) nor with disk diffusion (120 µg/disc) methods.

Since this is a first report of antimicrobial activity of fractionated organic extracts of *A. rigida,* there are no directly comparable data in reported literature. Only one study recently evaluated this species of red alga collected from the Indian Ocean but specifically focused on its polysaccharides extracts tested against only one isolate of a multidrug resistant *Salmonella typhi* [15]. That study also employed both disk diffusion and broth microdilution tests and reported a very strong antibacterial activity that exceeded the inhibition observed with streptomycin, which the authors argued to be associated with the sulfur-groups of the polysaccharides. Moreover, the study optimized the extraction of polysaccharides achieving high extraction yields so the compound(s) responsible for the observed antibacterial activity are different than present in two fractions (F3 and F4) of the present study. Two other studies evaluated the antimicrobial activity of unspeciated *Amphiroa* algae. One was conducted in the Gulf of Thailand and reported a very weak activity (zone of inhibition of less than 1 mm) of the aqueous extract against *S. aureus*, *Proteus mirabilis* and *C. albicans*, but no activity of the ethanolic extract [17]. The other study was conducted in India and tested methanolic extract against three *Aspergillus* spp. that were all mild to moderately inhibited by the disk diffusion method. Another study of methanolic extracts of *A. bowerbankii* and *A. ephedraea* algae from the east coast of South Africa reported relatively high minimal inhibitory concentrations of 6.25 mg mL^−1^ (*w*/*v*) against both *S. aureus* and *B. subtilis* [18]. Interestingly, that study used bimonthly sampling and reported seasonal variation of antibacterial activity that was observed only in late autumn and winter against *B. subtilis* and bimodally in autumn and spring against *S. aureus*. The reasons for seasonal effects and its ecological function have not been elucidated but have been observed in other studies. For instance, two studies of another red alga *Asparagopsis taxiformis* collected near Sicily, Italy showed antibacterial activities only in colder months of the year; March to May vs. June to September [67,68]. If the single-point sampling in September of this study has similarly affected the lack of antimicrobial activity of *A. rigida* from the Adriatic Sea remains to be further investigated.

### 2.5. Antioxidant Activity of A. rigida

In the present study, different polarity fractions were first analyzed for their total phenolic content since it is well known that phenolics exhibit antioxidant properties [69]. As can be seen in Table 5, the distribution of phenolic compounds in *A. rigida* demonstrated that among two less polar fractions, F3 contained the higher amount, 59.38 ± 1.62 mg GAE per gram of fraction, than fraction F4 (16.71 ± 0.29 mg g^−1^ GAE). In comparison, other studies evaluating the total phenolic content of red algae reported the values of 10.7 ± 0.3 and 4.7 ± 0.6 mg g^−1^ GAE for the methanolic extract from *Palmaria palmate* and *Porphyra tenera* [70], respectively, while for the two other red algae (*Gracilaria opuntia*, and *Digenea simplex*) TPC was on average around 31.03 mg g^−1^ GAE [66], indicating the higher polyphenolic content in *A. rigida*.

**Figure 1:**
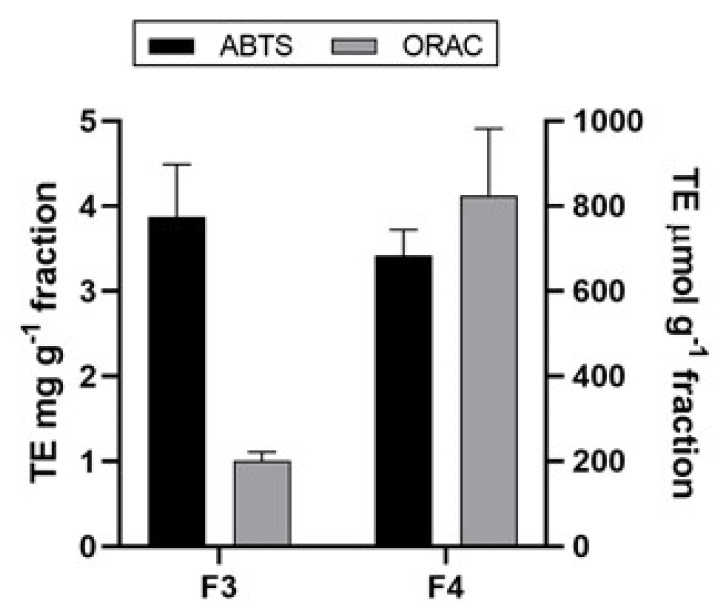
Antioxidant activities using diammonium salt of 2,2′-azino-bis (3-ethylbenzthiazolin-6-yl) sulfonic acid (ABTS) and oxygen radical absorbance capacity (ORAC) assays of the polarity fractions from *A. rigida*. The values are mean values ± standard deviations (*n* = 3).

The literature reports on a correlation between phenolic content and antioxidant activity are already present [71,72]. The antioxidant activity of polyphenols is the result of their ability to act as reducing agents, hydrogen donors and free radical quenchers, or even as metal chelators [73]. However, other compounds such as pigments (i.e., carotenoids), polysaccharides, proteins or peptides can also influence the antioxidant activity [71]. Since antioxidants are usually involved in several mechanisms of action, a single assay could not accurately reflect all of the antioxidants in a complex fraction. Therefore, it is necessary to use at least two different methods to evaluate the antioxidant activity of obtained fractions [74,75]. Herein, the antioxidant activity of the fractions F3 and F4 of *A. rigida* were tested by implementing two antioxidant assays—ABTS and ORAC. The results obtained using ABTS and ORAC are depicted in Figure 1. ABTS assay is based on the inhibition of the absorbance (decolorization of the ABTS^•+^, through measuring the reduction of the radical cation). The results of ABTS measurement implied relatively low antioxidant activity for all tested fractions with the highest inhibition percentage around 30% for F3 (5 mg mL^−1^) and presented as 3.87 ± 0.62 mg g^−1^ trolox equivalents (TE), while for F4 the activity was 3.42 ± 0.31 mg g^−1^ TE. The ORAC assay measures a fluorescent signal from a probe that is quenched in the presence of reactive oxygen species (ROS). The addition of an antioxidant absorbs the generated ROS, allowing the fluorescent signal to persist. The results of the ORAC assay within this study revealed that the highest activity was obtained for F4 (825.63 ± 154.45 μmol g^−1^ TE) followed by F3 (205.11 ± 27.21 μmol g^−1^ TE). Considering the polarity of these fractions, the obtained order could be ascribed to the highest carotenoid content in the least polar fraction F4, as it was presented in Table 4 for F4 where α-carotene and β-carotene were detected for which is known that they possess a significant antioxidant activity [76,77]. It was also reported that the pigments such as lutein and astaxanthin have major contribution to the antioxidant activity of macroalgae [2]. It is evident that the antioxidant activity of fractions of *A. rigida* is dependent not only on polyphenols but also other compounds that exhibit different antioxidant modes of action such as carotenoids.

## 3. Materials and Methods

### 3.1. Chemicals

The fibers covered with PDMS/DVB (polydimethylsiloxane/divinylbenzene) or DVB/CAR/PDMS (divinylbenzene/carboxen/polydimethylsiloxane) that were used for HS-SPME were purchased from Supelco Co. (Bellefonte, PA, USA).

The fatty acids methyl esters (FAMEs) standard used for determination of the fatty acid profile of *A. rigida* was purchased from Supelco Co. (Bellefonte, PA, USA).

HPLC standards of analyzed pigments were purchased from Sigma-Aldrich (St. Louis, MI, USA) including α-carotene (purity ≥ 97%), chlorophyll *a* (purity ≥ 95%), lutein (purity ≥ 96%) and fucoxanthin (purity ≥ 97%) while β-carotene (purity ≥ 95%) was purchased from Dr. Ehrenstorfer (Augsburg, Germany).

Mueller–Hinton agar was purchased from Merck (Darmstadt, Germany), YPD agar was purchased from Sigma Aldrich (St. Louis, MI, USA), bacteriological agar was purchased from Biolab (Budapest, Hungary) and Zobell Marine agar was purchased from HiMedia (Mumbai, India). Antimicrobial agent norfloxacin and florfenicol were purchased from Sigma Aldrich (St. Louis, MI, USA) and nystatin was purchased from Acros Organics (Geel, Belgium).

Folin–Ciocalteu reagent and sodium bicarbonate solution were purchased from Kemika (Zagreb, Croatia), the standards of gallic acid (>97.5%), ABTS (>99%), DCF (90%) and AAPH (97%) were purchased from Sigma Aldrich (St. Louis, MI, USA).

All used solvents were of HPLC grade and they were purchased from J.T. Baker (New Jersey, PA, USA). Other used chemicals were of analytical grade.

### 3.2. Material and Preparation Procedure

The sample of *Amphiora rigida* J.V. Lamouroux was collected in the middle part of the Adriatic Sea coast, close to Zadar (Šepurine), in September 2020 (44°12′42″ N; 15°09′23″ E). Single point sample collection provided representative sample. The alga was collected from depths of 2 m with a sea temperature at 20 °C. The samples were collected and placed in air tight plastic bags containing surrounding seawater and were immediately transported to the laboratory.

Prior to HS-SPME and HD, the samples were kept in the dark at 4 °C and the extractions were performed within 48 h of the collection. The alga was cut into small pieces and the excess seawater was removed by placing it between filter paper layers for 2 min (the seawater was not removed completely) as was done in previous research [21,22,23]. A part of the collected sample of *A. rigida* was subjected to air-drying at a room temperature in the dark for 14 days and used as dried material for HS-SPME and HD.

Furthermore, for the extraction of fatty acids and pigments performed by the procedures explained in Section 3.6 and Section 3.7, fresh *A. rigida* was freeze-dried before the extractions. For the freeze-drying experiment the sample was washed three times in demineralized water than it was cut in slices and frozen at −20 °C for 24 h in an ultra-low freezer. Three trays of frozen samples were placed in a laboratory freeze dryer (Martin Christ, Alpha 2–4 LSCplus, Osterode am Harz, Germany). The freeze drying process was performed for 96 h under high vacuum (0.5–1.81 hPa) with primary and secondary drying temperatures of −20 °C and 20 °C, respectively. Freeze-dried samples were further used for the determination of fatty acids and pigments content and for the evaluation of antimicrobial and antioxidant activities.

### 3.3. Headspace Solid-Phase Microextraction (HS-SPME)

Headspace solid-phase microextraction (HS-SPME) was performed with a manual SPME holder using two fibers covered with PDMS/DVB (polydimethylsiloxane/divinylbenzene, 65 μm) or DVB/CAR/PDMS (divinylbenzene/carboxen/polydimethylsiloxane, 50/30 μm). The fibers were conditioned prior to the extraction according to Supelco Co. Prepared samples (1 g) were placed separately in 5 mL glass vials and hermetically sealed with PTFE/silicone septa. The vials were maintained in a water bath at 60 °C during equilibration (15 min) and HS-SPME (45 min). After the sampling, the SPME fiber was withdrawn into the needle, removed from the vial and inserted into the injector (250 °C) of GC-FID and GC–MS for 6 min for thermal desorption directly to the GC column. The procedure was similar as in previous papers [21,22,23]. HS-SPME was done in triplicate.

### 3.4. Hydrodistillation (HD)

Hydrodistillation was performed in a modified Clevenger apparatus for 2 h with the use of 1 mL of solvent trap (pentane:diethyl ether 1:2 *v*/*v*). The fresh and air-dried samples (10 g; cut into small pieces) were used separately for the hydrodistillation. The volatile oil dissolved in the solvent trap was removed with a pipette, passed through the layer of MgSO_4_ in a small glass funnel and carefully concentrated by the slow flow of nitrogen until the volume of 0.2 mL. The hydrodistillation was performed in triplicate. 2 μL were used for GC-FID and GC–MS analyses.

### 3.5. Gas Chromatography (GC) Analysis

Gas chromatography and mass spectrometry (GC–MS) analyses were done on an Agilent Technologies (Palo Alto, CA, USA) gas chromatograph model 7890A equipped with a flame ionization detector (FID) and a HP-5MS capillary column (5% phenyl-methylpolysiloxane, Agilent J and W). The GC conditions were the same as described previously [21,22,23]. In brief, the injector and detector temperatures were 250 °C and 300 °C, the oven temperature was set up isothermal at 70 °C for 2 min, then increased from 70 to 200 °C at 3 °C min^−1^, and held isothermally at 200 °C for 15 min; split ratio was 1:50; carrier gas was helium (He at flow rate 1.0 mL min^−1^). The GC–MS analyses were done on an Agilent Technologies (Palo Alto, CA, USA) gas chromatograph model 7820A equipped with a mass selective detector (MSD) model 5977E (Agilent Technologies) and HP-5MS capillary column (30 m × 0.25 mm, 0.25 μm film thickness, Agilent Technologies, Palo Alto, CA, USA), under the same conditions as for the GC-FID analysis. The MSD (EI mode) was operated at 70 eV, and the mass range was 30–300 amu.

The identification of the compounds was based on the comparison of their retention indices (RI), determined relative to the retention times of *n*-alkanes (C_9_–C_25_), with those reported in the literature (National Institute of Standards and Technology [78]) and their mass spectra with the spectra from Wiley 9 (Wiley, New York, NY, USA) and NIST 17 (D-Gaithersburg) mass spectral libraries. The percentage composition of the samples was computed using the normalization method (without correction factors). The average component percentages in Table 1 and Table 2 were calculated from GC-FID and GC–MS analyses of three replicates.

### 3.6. Extraction and Fractionation with Solid-Phase Extraction (SPE)

The extraction was performed on freeze-dried sample at a room temperature with a mixture of solvents methanol (MeOH)/dichloromethane (DCM) (1:1, *v*/*v*) with the solvent:solid ratio 10 mL/g, three times for 5 min; each time with sonication (ultrasound-bath Elma, Elmasonic P 70 H, 37 kHz/50 W, Singen, Germany) and gravity filtration. The obtained extract was mixed with C18 powder (Macherey-Nagel Polygoprep 60-50 C18, 40–63 µm, FisherScientific, Massachusetts, USA) and the mixture was subjected to evaporation under nitrogen (5.0, Messer, Croatia) to remove the organic solvent. The dry extract mixed with C18 powder was then placed on the SPE cartridge (C18, 1 g, 6 mL, 40 µm; Agilent Bond Elut, Waldbronn, Germany), which was conditioned with MeOH and ultrapure H_2_O. The sample was eluted using the solvents of decreasing polarity: (F1) H_2_O, (F2) H_2_O/MeOH (1:1, *v*/*v*), (F3) MeOH and (F4) MeOH/DCM (1:1, *v*/*v)* (using 12 mL of each solvent). The water soluble components were eluted in F1 and F2 leading to better purification of the extract and obtaining higher amounts of compounds of interest (i.e., pigments) in F3 and F4. Fractions F3 and F4 were dried using SpeedVac (SPD1030, Thermo Scientific, Waltham, MA, USA) and stored at 4 °C in the dark prior to the analysis. The fractionation was performed in triplicates. Furthermore, less polar fractions F3 and F4 were used for pigment determination and for antimicrobial and antioxidant activities.

### 3.7. Extraction and GC-FID Analysis of Fatty Acids

Extraction of total lipids from *A. rigida* was performed by the Folch method [79]. Briefly, 1.00 g of the sample was mixed with 20 mL DCM/MeOH (2:1 *v*/*v*) solvent mixture. The mixture was stirred for 20 min at 400 rpm (IKA, KS 260 Basic, Staufen, Germany), filtered and then washed with 4 mL of 0.9% NaCl solution. The upper phase was removed, and lower chloroform phase containing lipids was evaporated in a rotary evaporator (Laborota 4010, Heidolph Instruments GmbH & Co. KG, Schwabach, Germany) at 60 °C. The samples were then dried in an oven (105 °C until constant weight). The extraction was performed in three repetitions. Afterwards, the fatty acids methyl esters (FAMEs) were prepared with cold methanolic potassium hydroxide solution according to the procedure described in Annex X B of the Commission Regulation No 796/2002 [80]. FAMEs were afterwards separated on a Shimadzu GC-2010 Plus gas chromatograph equipped with a flame ionization detector (FID) and fitted with a SH-Rtx-Wax capillary column (30 m, 0.25 mm i.d. and 0.25 µm thick stationary phase). Nitrogen was used as the carrier gas, flowing at the constant linear velocity of 1.33 mL min^−1^. The split/splitless injector was set at 250 °C, split ratio was 1:10, and the injection volume 2 µL. Initial column temperature of 110 °C was held for 2 min, then gradually increased 10 °C min^−1^ until temperature of 175 °C that was hold for 8 min, followed by gradual increase 5 °C min^−1^ until 210 °C held for 5 min, and a temperature increase to a final temperature of 230 °C by a rate of 5 °C min^−1^. Final temperature was held for 7 min. Total analysis time was 42.5 min. Flame ionization detector temperature was 300 °C. Hydrogen flow rate was 40 mL min^−1^, air flow rate 400 mL min^−1^ and make-up gas (nitrogen) flow was 30 mL min^−1^. Identification of separated FAMEs in samples was achieved based on the comparison of retention times with the retention times of certified reference standard (Supelco F.A.M.E. Mix, C_4_–C_24_) analyzed under the same conditions. The results were expressed as the percentage of identified fatty acid on total fatty acids (%).

### 3.8. High Performance Liquid Chromatography (HPLC) Analysis of Pigments

HPLC system, Agilent 1100 Series HPLC System (Agilent Technologies, Waldbronn, Germany) equipped with binary pump, autosampler, column heater and variable wavelength detector (VWD) was used for the separation, identification and quantification of chlorophylls and carotenoids from F3 and F4 obtained by SPE according to modified method by Castro-Puyana et al. [81]. Briefly, the YMC-C30 reversed phase column (250 mm × 4.6mm id, 5 µm particle size, YMC Europe, Schernbeck, Germany) was used and the mobile phases were: the mixture of MeOH:isopropanol:H_2_O (90:7:3, *v*/*v*/*v*) (A) and MeOH:isopropanol (10:90, *v*/*v*) (B) eluted according to the following gradient: 0 min, 0% B; 50 min, 100% B and 60 min, 0% B. The flow rate was 0–50 min, 0.6–0.8 mL/min and 50–60 min, 0.8 mL/min, the injection volume was 30 µL and detection was at 450 nm (recorded spectra from 350 to 660 nm by VWD). The identification of carotenoids and chlorophylls was carried out by comparing retention times and spectral data of separated peaks with those of standards and the quantification of pigments was performed by the external standard method. For the calibration curve, standard solutions of α-carotene (linearity R^2^ = 0.99189), β-carotene (linearity R^2^ = 0.98610), chlorophyll *a* (linearity R^2^ = 0.99934), lutein (linearity R^2^ = 0.99630) and fucoxanthin (linearity R^2^ = 0.99967) were prepared by the appropriate dilution of stocks of each standard in acetone. The analyses were performed in triplicates. The results in Table 4 were calculated as mean values ± standard deviation (SD) and expressed as mg g^−1^ of the dry fraction.

### 3.9. Antimicrobial Activity

The semipurified fractions (F3 and F4) were tested for antimicrobial activity using disk diffusion for qualitative and broth microdilution methods for quantitative assessment according to the CLSI guidelines [82,83] with minor modifications. All stock solutions were made at 20 mg mL^−1^ (*w*/*v*), where dry residues of F3 and F4 were diluted in the appropriate solvent. F3 was dissolved in MeOH and 50% (*v*/*v*) DMSO, while F4 was dissolved in 50% (*v*/*v*) DMSO. These stock solutions were diluted with distilled H_2_O to working solutions of 4 mg mL^−1^ (*w*/*v*), which were used for disk diffusion (120 µg/disc) tests using Mueller–Hinton agar for bacteria and YPD agar prepared from YPD broth supplemented with 1.5% (*w*/*v*) bacteriological agar for fungi. These working solutions were then further diluted with culture media broths (cation-adjusted Mueller–Hinton broth for bacteria and YPD broth for fungi) to 400–0.78 µg mL^−1^ (*w*/*v*) as final concentrations used in broth microdilution tests. All tests were performed on two independent occasions each time in technical duplicates. Each experimental run included the solvent control at the concentration corresponding to the samples (20% (*v*/*v*) MeOH and 10% (*v*/*v*) DMSO in disk diffusion and 2% (*v*/*v*) MeOH and 1% (*v*/*v*) DMSO in broth microdilution tests) and an antimicrobial agent norfloxacin for bacteria and nystatin for fungi. Broth microdilution tests additionally employed a positive growth control (drug- and sample-free wells) and a negative sterility control (wells with culture media only). The bacterial panel consisted of Gram negative (*Escherichia coli* NCTC 12,241 and *Pseudomonas aeruginosa* NCTC 12903) and Gram positive (*Staphylococcus aureus* ATCC 6538 and *Bacillus subtilis* subsp. *spizizenii* ATCC 6633) indicator species and the fungal panel of *Candida albicans* ATCC 90,028 yeast and *Fusarium oxysporum* f. sp. *lagenariae* JCM 9293 mold species. All tests were performed in aerobic atmosphere at 35 °C. In addition, field isolates of *Listonella* (*Vibrio*) *anguillarum*, *Photobacterium damselae* subsp. *damselae* and *P. damselae* subsp. *piscicida* isolated from diseased fish were tested by the disk diffusion method only. For these aquatic bacteria grown also aerobically but at 28 °C on Zobell Marine agar, the antimicrobial quality control range of *E. coli* NCTC 12,241 for florfenicol was used according to Miller et al. [84].

### 3.10. Antioxidant Scavenging Ability Determination

Obtained dry residues for F3 and F4 were dissolved in a proper solvent, following the polarity of fractions. Used solvent for fraction F3 was MeOH, while DMSO was used for F4. The stock concentration was highly dependable on the obtained mass of fractions, so the samples F3 and F4 were prepared at maximum concentration of 5 mg mL^−1^. Afterward, proper dilutions for each sample were made.

All spectrophotometric measurements were carried out using an UV/Vis microplate reader (Infinite M200 PRO, TECAN, Männedorf, Switzerland) in the multiwell plate (96-well) in triplicates for all used assays. Results for all assays are calculated as mean ± standard deviation (*n* = 3).

The Folin–Ciocalteu method was used for the total phenolic content (TPC) measurement [85] with some adaptations. Briefly, 100 μL extract was mixed with 750 μL of Folin–Ciocalteu reagent (previously diluted tenfold with distilled water) and allowed to stand at room temperature for 5 min. Next, 750 μL sodium bicarbonate solution (60 g L^−1^) was added to the mixture. After incubation for 90 min at room temperature, the absorbance was measured at 750 nm. Total phenolic content was calibrated against gallic acid standard and expressed as mg gallic acid equivalent (GAE) per g of biomass.

The antioxidant capacity was measured by two assays (ORAC and ABTS). For the implementation of the ABTS method and for comparison purposes, the traditional ABTS^+•^ radical-cation discoloration assay was used by means of spectroscopy at 734 nm [86]. The ABTS^+•^ stock solution was prepared from a 7 mM ABTS (diammonium salt of 2,2′-azino- bis (3-ethylbenzthiazolin-6-yl) sulfonic acid) with 2.45 mM of potassium persulfate dissolved in 5 mL of distilled water, allowed to react during 17 h at room temperature in the absence of light. The working solution of the already preformed ABTS^+•^ radical-cation was diluted with ethanol to achieve absorbance of 0.700 ± 0.02. The reaction mixture comprised of sample and ABTS^+•^ solution resulted in an inhibition between 20 and 80% of the blank of measurement. The blank was represented by used solvent for each fraction. Results were expressed in mg TE g^−1^ of fraction since trolox was used as an antioxidant standard.

For measuring the radical absorbance capacity, the oxygen radical absorbance capacity (ORAC) assay as described by Huang et al. [87] was used with slight modification. Different concentrations of each fraction were prepared using appropriate solvent MeOH for F3, and DMSO for F4, because different activity was expected for each fraction. A fixed volume of 25 μL of the diluted samples was loaded in black 96-well flat bottom plates. The same amount of trolox (6.25–100 μM) and solvents were used in the plates as standard and blanks, respectively. Then 150 μL of 2′,7′-dichlorofluorescein (DCF) solution (1:500, *v*/*v* in 25 mL 75 mM PBS) was added and the mixture was incubated at 37 °C for 30 min in an incubator with shaking (New Brunswick, Innova 42). Then 25 μL AAPH (2,2-Azobis (2-methylpropionamidine) dihydrochloride, 75.3 mM) was added to the mixture to start the reaction and the fluorescence was recorded every 10 min for 16 h (overnight kinetic cycle). Samples were assayed at an excitation wavelength of 485 nm and an emission wavelength of 528 nm with optimal fluorescence gain of 188. The oxygen radical absorbance capacity of the samples was expressed as μmol TE g^−1^ of fraction.

## 4. Conclusions

The headspace profile of *A. rigida* dominated by two normal odd alkanes heptadecane and pentadecane and the comparison of HS-FrAr and HS-DrAr chemical profiles pointed out many similarities. The dominance of heptadecane can be connected with the decomposition of stearic acid found in *A. rigida*. In addition, β-ionone found in HS-FrAr can be the degradation product of β-carotene that was present as the main carotenoid in *A. rigida*. The predominant compound in HD-FrAr was (*E*)-phytol accompanied with a smaller abundance of its degradation product phytone. It can be suggested that (*E*)-phytol was formed by the degradation of chlorophyll *a* found in *A. rigida*. Unlike the headspace profiles, hydrodistillate chemical profiles were quite different when HD-DrAr and HD-FrAr were compared. (*E*)-phytol was approximately one-half reduced, but the abundance of phytone as well of unsaturated and oxygenated compounds increased indicating more intense fatty acids decomposition.

The main fatty acids were palmitic acid, as the dominant one, followed by eicosapentaenoic acid (EPA) and stearic acid. The content of saturated fatty acids (SFAs) was almost double than the amount of polyunsaturated fatty acids (PUFAs). Given the relatively high content of EPA in this red alga, there is a possibility of using this alga as a good source of essential omega-3 fatty acids but further work for investigation of the bioavailability is necessary.

Along with β-carotene, lutein was also determined as the second major carotenoid, while fucoxanthin and α-carotene were determined as minor components.

According to the obtained results for antioxidant scavenging ability, it can be suggested that the carotenoids play a major role in the antioxidant activity of *A. rigida*.

Overall, the present study contributes toward better chemical characterization of marine red alga *Amphiroa rigida* J.V. Lamouroux in general and to better understanding of marine algal biodiversity in the Adriatic Sea.

## Figures and Tables

**Table 1:** The headspace organic compounds of *A. rigida* determined by HS-SPME/GC–FID/MS (I—fresh sample extracted by DVB/CAR/PDMS fiber; II—fresh sample extracted by PDMS/DVB fiber; III—air-dried sample extracted by DVB/CAR/PDMS fiber and IV—air-dried sample extracted by PDMS/DVB fiber).

No	Compound	RI	I Av ± SD	II Av ± SD	III Av ± SD	IV Av ± SD
1.	Dimethyl sulfide	<900	-	-	4.38 ± 0.14	1.22 ± 0.02
2.	Pentanal ^S^	<900	1.25 ± 0.03	0.79 ± 0.03	1.84 ± 0.05	0.68 ± 0.01
3.	(*E*)-Pent-2-enal ^S^	<900	0.53 ± 0.02	-	-	-
4.	Pentan-1-ol ^S^	<900	0.67 ± 0.02	0.86 ± 0.03	-	-
5.	(*Z*)-Pent-2-en-1-ol ^S^	<900	1.22 ± 0.03	-	-	-
6.	Hexanal ^S^	<900	6.72 ± 0.10	2.31 ± 0.02	6.31 ± 0.21	2.01 ± 0.02
7.	(*E*)-Hex-2-enal ^S^	<900	7.41 ± 0.23	0.48 ± 0.03	2.47 ± 0.04	0.87 ± 0.03
8.	Hexan-1-ol ^S^	<900	2.36 ± 0.02	1.09 ± 0.03	-	-
9.	Tribromomethane ^S^	<900	1.52 ± 0.04	1.27 ± 0.02	-	-
10.	Heptanal ^S^	901	0.49 ± 0.02	0.47 ± 0.02	1.00 ± 0.03	0.48 ± 0.01
11.	Benzaldehyde ^S^	967	0.93 ± 0.03	1.60 ± 0.04	1.16 ± 0.05	1.04 ± 0.03
12.	Oct-1-en-3-ol ^S^	982	0.50 ± 0.03	0.40 ± 0.01	0.51 ± 0.03	0.47 ± 0.01
13.	Octane-2,3-dione	984	-	-	1.21 ± 0.05	0.80 ± 0.03
14.	6-Methylhept-5-en-2-one ^S^	988	0.67 ± 0.03	0.44 ± 0.03	0.90 ± 0.02	0.71 ± 0.02
15.	2-Pentylfuran ^S^	993	-	-	0.98 ± 0.03	0.80 ± 0.04
16.	Octanal ^S^	1003	0.53 ± 0.02	0.74 ± 0.02	-	-
17.	(*E*,*E*)-Hepta-2,4-dienal ^S^	1014	-	0.14 ± 0.01	-	-
18.	Benzyl alcohol ^S^	1042	-	-	8.58 ± 0.10	9.44 ± 0.23
19.	(*E*)-Oct-2-enal ^S^	1061	0.69 ± 0.03	0.48 ± 0.05	0.13 ± 0.03	0.44 ± 0.05
20.	(*E*,*E*)-Octa-3,5-dien-2-one	1073	-	-	-	1.13 ± 0.04
21.	Octan-1-ol ^S^	1074	0.34 ± 0.05	0.49 ± 0.02	-	-
22.	Nonanal ^S^	1099	1.27 ± 0.05	1.63 ± 0.03	0.99 ± 0.02	1.50 ± 0.05
23.	6-[(1*Z*)-1-Butenyl]-cyclohepta-1,4-diene (Ectocarpene)	1150	0.97 ± 0.02	0.66 ± 0.05	-	-
24.	Decanal ^S^	1296	-	-	-	0.62 ± 0.03
25.	β-Cyclocitral ^S^	1222	0.78 ± 0.05	0.47 ± 0.02	-	0.67 ± 0.01
26.	(*E*)-Dec-2-enal ^S^	1264	-	0.16 ± 0.02	-	-
27.	α-Cubebene	1366	-	0.88 ± 0.03	-	-
28.	β-Bourbonene	1385	-	0.37 ± 0.02	0.90 ± 0.02	1.53 ± 0.02
29.	Germacrene D ^S^	1481	-	1.13 ± 0.05	1.33 ± 0.06	2.66 ± 0.05
30.	(*E*)-β-Ionone ^S^	1486	1.55 ± 0.04	0.79 ± 0.04	0.50 ± 0.02	0.53 ± 0.02
31.	Pentadec-1-ene ^S^	1492	1.10 ± 0.06	13.05 ± 0.24	-	-
32.	(*E*)-Pentadec-7-ene *	1495	-	6.00 ± 0.32	-	-
33.	Pentadecane ^S^	1500	5.51 ± 0.25	7.08 ± 0.20	5.21 ± 0.10	7.02 ± 0.23
34.	Hexadec-7-ene *	1515	-	11.25 ± 0.22	-	-
35.	Ttradecan-1-ol ^S^	1678	0.96 ± 0.05	1.02 ± 0.06	-	-
36.	Heptadec-1-ene ^S^	1692	0.62 ± 0.05	1.04 ± 0.06	-	-
37.	(*E*)-Heptadec-8-ene	1678	0.76 ± 0.05	0.77 ± 0.03	0.10 ± 0.02	0.28 ± 0.02
38.	Heptadecane ^S^	1700	47.77 ± 2.66	30.08 ± 1.67	50.16 ± 3.05	53.96 ± 4.05
39.	(*E*)-Heptadec-3-ene *	1717	3.63 ± 0.05	4.79 ± 0.06	6.44 ± 0.25	7.41 ± 0.45
40.	Eicosane ^S^	2000	-	0.58 ± 0.03	-	-

Av—average area percentage composition obtained by GC-FID/MS of 3 replicates, SD—standard deviation of the area percentages for 3 replicates, RI—retention indices relative to C_9_–C_25_ alkanes, *—tentatively identified, ^S^—identification confirmed with standard compound.

**Table 2:** The volatile oil compositions of *A. rigida* determined by HD/GC–MS (I—volatile oil composition from fresh sample and II—volatile oil composition from air-dried sample).

No	Compound	RI	I Av ± SD	II Av ± SD
1.	(*E*)-Hex-2-enal ^S^	<900	0.85 ± 0.02	1.55 ± 0.03
2.	Heptan-2-one ^S^	<900	-	0.12 ± 0.01
3.	Heptanal ^S^	901	0.04 ± 0.01	0.12 ± 0.01
4.	(*E*)-Hept-2-enal ^S^	961	-	0.05 ± 0.01
5.	Benzaldehyde ^S^	967	0.26 ± 0.02	0.31 ± 0.02
6.	Oct-1-en-3-ol ^S^	982	-	0.09 ± 0.01
7.	Octan-2,3-dione	984	-	0.21 ± 0.02
8.	6-Methylhept-5-en-2-one ^S^	988	0.50 ± 0.02	0.36 ± 0.02
9.	Octan-2-one ^S^	992	0.05 ± 0.03	0.25 ± 0.04
10.	Octanal ^S^	1003	0.12 ± 0.02	0.21 ± 0.07
11.	(*E*,*E*)-Hepta-2,4-dienal ^S^	1014	-	0.12 ± 0.02
12.	2-Ethylhexan-1-ol ^S^	1033	0.12 ± 0.02	0.12 ± 0.01
13.	2,6,6-Trimethylcyclohexanone	1040	-	0.03 ± 0.01
14.	Benzyl alcohol ^S^	1042	0.26 ± 0.02	0.23 ± 0.01
15.	Phenylacetaldehyde ^S^	1049	0.14 ± 0.02	0.17 ± 0.02
16.	(*E*)-Oct-2-enal ^S^	1061	0.08 ± 0.02	0.22 ± 0.03
17.	Acetophenone ^S^	1072	-	0.08 ± 0.01
18.	Octan-1-ol ^S^	1074	0.16 ± 0.02	0.23 ± 0.03
19.	Nonan-2-one ^S^	1091	0.05 ± 0.02	0.06 ± 0.01
20.	(*E*,*Z*)-Octa-3,5-dien-2-one	1095	-	0.06 ± 0.01
21.	Nonanal ^S^	1099	0.10 ± 0.02	0.23 ± 0.04
22.	(*E*,*E*)-Octa-2,4-dienal	1111	-	0.18 ± 0.02
23.	Phenylacetonitrile ^S^	1144	0.08 ± 0.01	0.01 ± 0.00
24.	4-Ketoisophorone ^S^	1148	0.04 ± 0.01	-
25.	6-[(1*Z*)-1-Butenyl]-cyclohepta-1,4-diene (Ectocarpene)	1150	0.01 ± 0.00	-
26.	(*E*,*Z*)-Nona-2,6-dienal ^S^	1157	-	0.12 ± 0.01
27.	(*Z*)-Non-2-enal ^S^	1163	-	0.12 ± 0.02
28.	3-Methylacetophenone ^S^	1187	-	0.06 ± 0.01
29.	Decan-2-one ^S^	1194	0.18 ± 0.02	0.48 ± 0.05
30.	Decanal ^S^	1296	-	0.16 ± 0.02
31.	β-Cyclocitral ^S^	1222	-	0.16 ± 0.03
32.	Benzothiazole ^S^	1227	0.10 ± 0.02	0.08 ± 0.01
33.	β-Cyclohomocitral	1260	-	0.14 ± 0.02
34.	(*E*)-Dec-2-enal ^S^	1264	-	0.16 ± 0.023
35.	Decan-1-ol ^S^	1276	-	0.21 ± 0.02
36.	Undecan-2-one ^S^	1294	0.04 ± 0.01	-
37.	(*E*,*Z*)-Deca-2,4-dienal	1294	-	0.14 ± 0.02
38.	Undecanal ^S^	1307	-	0.17 ± 0.01
39.	(*E*,*E*)-Deca-2,4-dienal ^S^	1318	-	0.31 ± 0.03
40.	Undecan-1-ol ^S^	1377	-	0.18 ± 0.02
41.	β-Bourbonene	1385	0.05 ± 0.01	0.03 ± 0.01
42.	β-Cubebene	1391	-	0.09 ± 0.02
43.	Tetradecane ^S^	1400	0.05 ± 0.01	-
44.	Dodecanal ^S^	1409	-	0.36 ± 0.05
45.	α-Ionone ^S^	1429	-	0.13 ± 0.06
46.	(*E*)-Geranylacetone ^S^	1455	-	0.85 ± 0.04
47.	Dodecan-1-ol ^S^	1477	0.44 ± 0.02	2.11 ± 0.20
48.	Germacrene D ^S^	1481	1.01 ± 0.06	0.46 ± 0.07
49.	(*E*)-β-Ionone ^S^	1486	0.29 ± 0.02	2.55 ± 0.06
50.	Pentadec-1-ene ^S^	1492	0.50 ± 0.02	0.39 ± 0.01
51.	(*E*)-Pentadec-7-ene *	1495	0.04 ± 0.01	-
52.	Pentadecane ^S^	1500	2.36 ± 0.05	0.91 ± 0.02
53.	*N*,*N*-Dimethyldodecan-1-amine	1504	0.94 ± 0.02	-
54.	Tridecanal ^S^	1510	-	0.47 ± 0.05
55.	Cubebol	1516	0.59 ± 0.03	0.57 ± 0.01
56.	Tridecan-1-ol	1578	0.29 ± 0.01	0.63 ± 0.01
57.	Hexadecane ^S^	1600	-	0.23 ± 0.02
58.	Tetradecanal ^S^	1612	-	0.48 ± 0.02
59.	Benzophenone ^S^	1627	-	0.66 ± 0.03
60.	Cubenol	1644	0.42 ± 0.02	1.95 ± 0.05
61.	Tetradecan-1-ol ^S^	1678	0.48 ± 0.02	2.76 ± 0.09
62.	Eudesma-4(15),7-dien-1β-ol	1686	1.06 ± 0.03	0.29 ± 0.02
63.	Heptadec-1-ene ^S^	1692	0.75 ± 0.02	0.82 ± 0.03
64.	(*E*)-Heptadec-8-ene ^S^	1678	0.31 ± 0.02	2.02 ± 0.03
65.	Heptadecane ^S^	1700	3.80 ± 0.21	12.22 ± 0.23
66.	Pentadecanal ^S^	1714	0.50 ± 0.02	1.07 ± 0.06
67.	(*E*)-Heptadec-3-ene *	1717	0.29 ± 0.02	2.50 ± 0.06
68.	*trans*-Farnesol ^S^	1724	0.10 ± 0.01	0.29 ± 0.02
69.	(*E*)-2-Hexylcinnamaldehyde	1748	-	0.25 ± 0.04
70.	Pentadecan-1-ol ^S^	1780	0.18 ± 0.02	0.95 ± 0.03
71.	2-Ethylhexyl salicylate	1805	0.64 ± 0.02	1.03 ± 0.09
72.	Octadecane ^S^	1800	0.22 ± 0.02	0.23 ± 0.02
73.	6,10,14-Trimethylpentadecan-2-one (Phytone)	1845	2.21 ± 0.09	7.28 ± 0.12
74.	(*Z*)-Hexadec-11-en-1-ol	1861	-	2.02 ± 0.10
75.	Diisobutyl phthalate ^S^	1868	0.55 ± 0.02	0.47 ± 0.03
76.	Hexadecan-1-ol ^S^	1881	1.13 ± 0.09	3.79 ± 0.80
77.	Nonadecane ^S^	1900	-	0.22 ± 0.02
78.	Heptadecan-2-one ^S^	1900	0.08 ± 0.01	0.22 ± 0.02
79.	(*E*,*E*)-Farnesyl acetone	1918	-	1.15 ± 0.08
80.	Methyl palmitate ^S^	1926	-	0.57 ± 0.02
81.	Dibutyl phthalate ^S^	1962	0.94 ± 0.04	1.65 ± 0.05
82.	Cyclooctasulfur	2009	0.22 ± 0.02	-
83.	Octadecanal ^S^	2019	-	0.87 ± 0.02
84.	Epimanool	2051	3.18 ± 0.09	1.77 ± 0.08
85.	(*E*)-Phytol ^S^	2112	41.75 ± 1.87	16.44 ± 0.15
86.	Pachydictyol A	2123	2.97 ± 0.09	1.11 ± 0.04
87.	Isopachydictyol A	2136	2.41 ± 0.02	1.75 ± 0.03
88.	Tricosane ^S^	2300	-	0.69 ± 0.02
89.	Ferruginol	2331	1.45 ± 0.023	-

Av—average area percentage composition obtained by GC-FID/MS of 3 replicates, SD—standard deviation of the area percentages for 3 replicates, RI—retention indices relative to C_9_–C_25_ alkanes, *—tentatively identified, ^S^—identification confirmed with standard compound.

**Table 3:** Fatty acids composition of *A. rigida* determined by GC-FID.

No.	Fatty Acid	Av ± SD * (%)
1.	Butanoic acid (Butyric acid) (C4:0)	0.20 ± 0.02
2.	Hexanoic acid (Caproic acid) (C6:0)	0.91 ± 0.08
3.	Tetradecanoic acid (Myristic acid) (C14:0)	3.34 ± 0.15
4.	Pentadecanoic acid (Pentadecyclic acid) (C15:0)	0.69 ± 0.01
5.	Hexadecanoic acid (Palmitic acid) (C16:0)	42.86 ± 0.26
6.	Octadecanoic acid (Stearic acid) (C18:0)	11.65 ± 0.10
	**Total saturated fatty acids (SFA)**	**59.65**
7.	(*Z*)-Hexadec-9-enoic acid (Palmitoleic acid) (C16:1)	1.73 ± 0.09
8.	(*Z*)-Octadec-9-enoic acid+(*E*)-Octadec-9-enoic acid (*cis*-Oleic acid+*trans*-Oleic acid) (C18:1n9c+t)	5.46 ± 0.03
	**Total monounsaturated fatty acids (MUFA)**	**7.19**
9.	(9*Z*,12Z)-Octadeca-9,12-dienoic acid (*cis*-Linoleic acid) (C18:2n6c)	3.03 ± 0.03
10.	(9*E*,12*E*)-Octadeca-9,12-dienoic acid (*trans*-Linoleic acid) (C18:2n6t)	2.22 ± 0.02
11.	(6*Z*,9*Z*,12*Z*)-Octadeca-6,9,12-trienoic acid (γ-Linolenic acid) (C18:3n6)	0.46 ± 0.05
12.	(9*Z*,12*Z*,15*Z*)-Octadeca-9,12,15-trienoic acid (α-linolenic acid) (C18:3n3)	0.41 ± 0.06
13.	(11*Z*,14*Z*)-Icosa-11,14-dienoic acid (Eicosadienoic acid) (C20:2n6)	7.90 ± 0.11
14.	(5*Z*,8*Z*,11Z,14*Z*,17*Z*)-Icosa-5,8-11,14,17-pentaenoic acid (Eicosapentaenoic acid) (C20:5n3)	19.14 ± 0.32
	**Total polyunsaturated fatty acids (PUFA)**	**33.16**
	**Total ω3 fatty acids**	**21.96**
	**Total ω6 fatty acids**	**13.61**

* Av—average area of 3 replicates expressed in percentage (%) with standard deviation (SD).

**Table 4:** The pigment composition of *A. rigida* determined by HPLC.

Pigments	F3 Av ± SD *	F4 Av ± SD *
Fucoxanthin	0.63 ± 0.62	n.d.
Lutein	5.83 ± 0.97	n.d.
Chlorophyll *a*	n.d.	13.65 ± 0.81
β-Carotene	n.d.	6.18 ± 0.72
α-Carotene	n.d.	n.q.

* Av—average area of 3 replicates expressed in mg g^−1^ of a dry fraction with standard deviation (SD); n.d.—not detected; n.q.—not quantified.

**Table 5:** Total phenolic content of red algae *A. rigida* crude fractions (Av ± SD; *n* = 3) expressed in mg GAE per g of dry fraction.

Fraction	mg GAE g^−1^ of Dry Fraction
F3	59.38 ± 1.62
F4	16.71 ± 0.29

## Data Availability

The data presented in this study are available for limited time on request from the corresponding author.

## Sample Availability

The sample of the alga is available from the authors for limited time.
